# Prenatal Diagnosis of Turner Syndrome Mosaicism: A Case Report

**DOI:** 10.7759/cureus.71371

**Published:** 2024-10-13

**Authors:** Isaac Abulatan, Nnenna J Mbara, Adegbenro O Fakoya

**Affiliations:** 1 Obstetrics and Gynecology, Pediatrics and Medicine, University of Medicine and Health Sciences, Basseterre, KNA; 2 Cellular Biology and Anatomy, Louisiana State University Health Sciences Center, Shreveport, USA

**Keywords:** gonadoblastoma, mosaicism, mosaic turner syndrome, multicystic kidney, non-disjunction

## Abstract

Turner syndrome is a chromosomal disorder affecting females characterized by the partial or complete absence of one X chromosome. The pathogenesis of Turner syndrome primarily arises from chromosomal nondisjunction during gametogenesis, leading to various genotypic presentations. The most common genotype is 45, XO, representing a monosomy of the X chromosome. However, mosaicism, where different cell lines carry distinct chromosomal compositions, is also observed in some cases. This paper presents a case of prenatal diagnosis of Turner syndrome mosaicism with a genotype of 45, XO/46, XY. The patient, initially managed by her obstetrician/gynecologist, was referred to a perinatologist at 20 weeks and four days of gestation due to multicystic kidney findings in the fetus. Initial non-invasive prenatal testing (NIPT) suggested trisomy 22, but confirmation through amniocentesis revealed mosaic Turner syndrome (45, XO/46, XY). The pregnancy culminated in the delivery of a phenotypically female infant at 39 weeks gestation. Mosaic 45, XO/46, XY presents with a broad spectrum of phenotypic variability, ranging from classic Turner syndrome features to ovotesticular disorder and genital ambiguity, and even male genitalia with infertility. Patients with mosaic Turner syndrome require multidisciplinary follow-up due to the complexity of their condition. Additionally, the presence of Y chromosome material significantly elevates the risk of gonadoblastoma, necessitating consideration of prophylactic gonadectomy.

## Introduction

Turner syndrome is a chromosomal abnormality affecting females where the X chromosome is partially or completely absent. It has a prevalence of 1:2000 to 1:3000 live female births [1]. The true prevalence is difficult to ascertain because milder phenotypes go undiagnosed, while some get diagnosed late in adulthood. The most common characteristic among Turner syndrome patients is short stature. Other characteristics include gonadal agenesis, webbed neck, cubitus valgus, cardiac malformations, renal anomalies, and other specific dysmorphic features. Most cases of Turner syndrome do not make it to birth, especially the 45 XO monosomy. It is estimated that 10% of spontaneous abortions are due to 45, XO monosomy [2].

The genotypic construct of Turner syndrome can happen in various ways, with the most common mechanism being chromosomal nondisjunction. The most common karyotype of Turner syndrome, which is 45, XO, occurs due to chromosomal nondisjunction during the formation of reproductive cells. Another karyotype associated with Turner syndrome is 45, XO, which may present with varying degrees of mosaicism. Mosaic Turner syndrome arises from the nondisjunction of sex chromosomes during early fetal development; one of the typical mosaics is 45, X/46, XX [2]. This mosaic pattern involves a mixture of cells with a single X chromosome (45, X) and cells with a typical female chromosomal complement (46, XX). Individuals with mosaics may exhibit milder phenotype features than those with complete X chromosome monosomy. Other rare mosaics include patterns such as 45, X/47, XXX and 45, X/47, XXY, and 45, X/48, XXXX, among others. These mosaics result from additional chromosomal anomalies beyond the typical 45, X pattern, leading to diverse clinical presentations and challenges in diagnosis and management [2].

Another chromosomal possibility in Turner syndrome is 45, X/46, XY. In this pattern, individuals have a combination of cells with a single X chromosome (45, X) and cells with a male chromosomal complement (46, XY). A 45, X/46, XY turner mosaic can have a broad spectrum of clinical manifestations, including ambiguous genitalia, gonadal dysgenesis, and varying degrees of virilization [2]. Not all 45, X/46, and XY karyotypes present as Turner syndrome. Mosaicism involving the 45, X/46, XY karyotype is associated with a diverse range of phenotypic outcomes. These can include the characteristic features of Turner syndrome, an ovotesticular disorder of sex development (DSD) presenting with ambiguous genitalia, and, in some cases, a normal male appearance accompanied by infertility. Each mosaic pattern of Turner syndrome presents unique clinical features and requires tailored diagnostic and therapeutic interventions to address specific needs and optimize outcomes for affected individuals. This report examines a case of Turner syndrome with a karyotype of 45, XO/46, XY to shed light on the intricacies of mosaic Turner syndrome. The clinical presentation, diagnostic approach, and therapeutic considerations are examined.

## Case presentation

A 30-year-old female patient, G3P2, was referred to the Maternal Fetal Medicine (MFM) clinic at 20 weeks four days of pregnancy for an enlarged multicystic right kidney in the fetus. The patient had been under the care of a regular obstetrician-gynecologist early in the pregnancy, with no notable complications or events reported during that time. She had a normal nuchal translucency (NT) test at 11 weeks five days but denied noninvasive prenatal testing (NIPT). The patient underwent an anatomical survey at 20 weeks and four days in the second trimester. An anatomical survey at that time revealed an active fetus in a transverse position with the fetal right kidney appearing enlarged and multicystic, for which she was referred to the MFM. The left kidney and bladder of the fetus were normal at this time, along with a normal amniotic fluid index (AFI).

**Figure 1:**
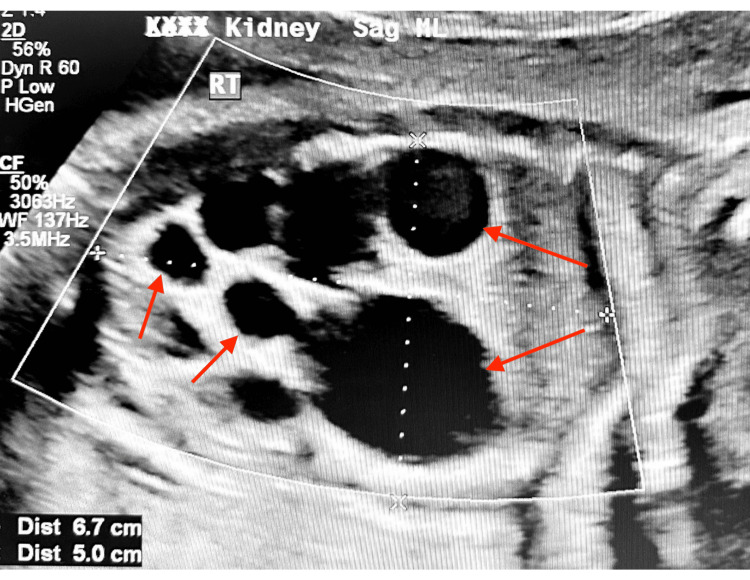
Fetal ultrasound scan of the kidney at 24 weeks, one day of gestation Arrows show the enlarged right multicystic kidney with the largest cyst measuring 5.3 x 3.2 x 2.9 cm.

The patient was seen at the MFM clinic at 22 weeks. An ultrasound was performed which revealed that the estimated fetal weight (EFW) was 16% and that the abdominal circumference (AC) was at the 19th percentile. AFI and maximal vertical pocket (MVP) were normal at this time. The fetal right kidney was also shown to be enlarged with numerous cysts, for which the largest cyst measured 0.8 x 0.8 x 1.2 cm. Umbilical artery doppler was normal at this time. A fetal echo was also performed, which was normal, with no abnormalities in cardiac structures noted. The MFM referred her to pediatric urology for the multicystic kidney. MFM also advised the patient for NIPT and maternal serum alpha-fetoprotein screening (MSAFP). She was also counseled to increase water and protein drinks. The result for NIPT showed trisomy 22. The possibility of amniocentesis for confirmation was discussed with the patient since NIPT is primarily a screening test. At 24 weeks and one day, the patient returned for amniocentesis as well as an ultrasound scan. The right kidney of the fetus was still enlarged with numerous cysts. The largest cyst measured 5.3 x 3.2 x 2.9 cm. Figure 1 shows a picture of the multicystic right kidney. Ambiguous genitalia were suspected at this time.

**Figure 2:**
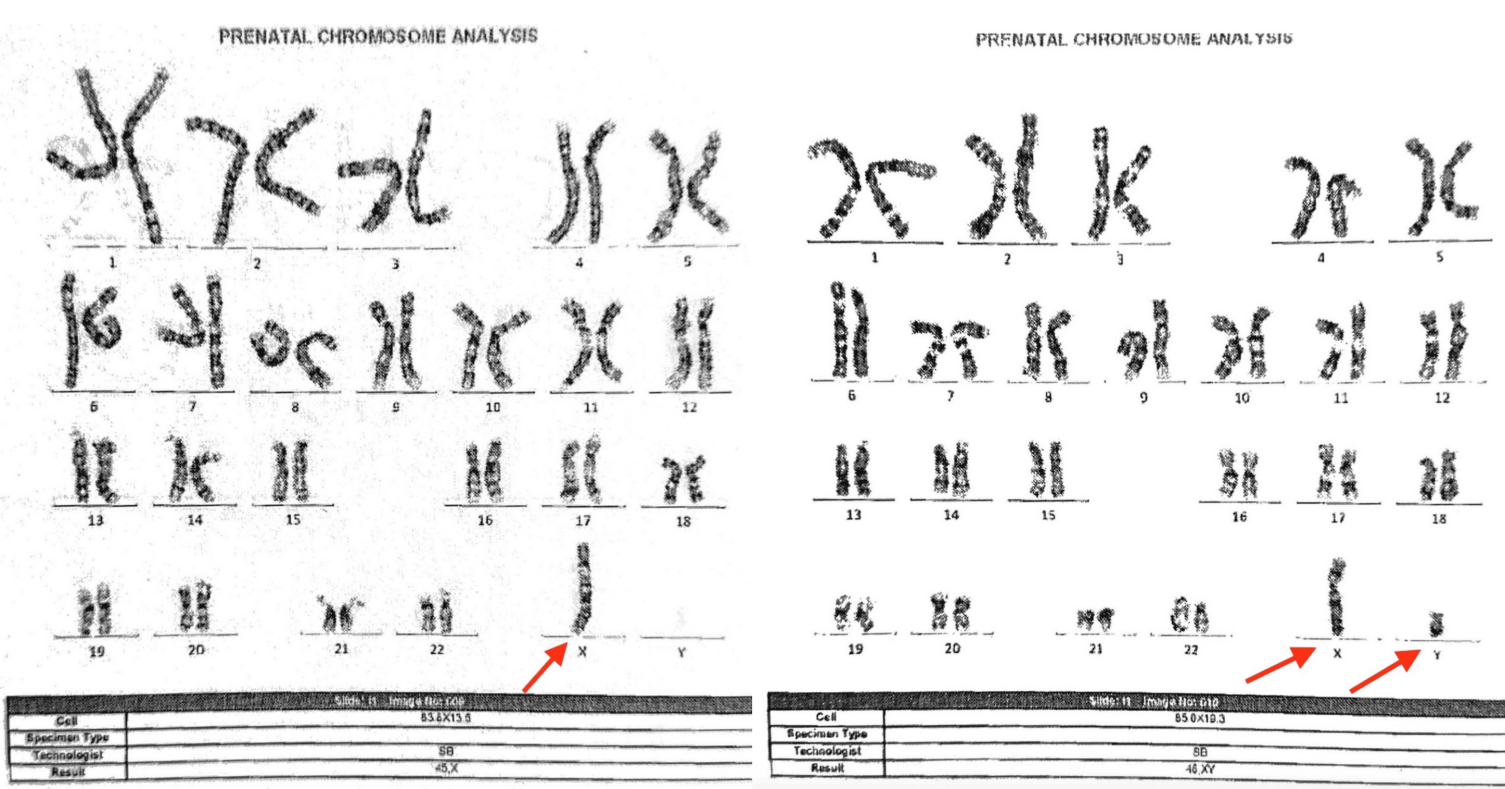
Karyotype analysis of amniotic fluid showing 45, X0, and 46, XY mosaicism

Amniotic fluid was sent for fluorescence in situ hybridization (FISH), karyotyping, and alpha-fetoprotein testing. Karyotype results, as shown in Figure 2, showed 45, XO/46, XY Turner syndrome mosaicism. The patient had a negative AFP result, and the amniocentesis result did not show trisomy 22.

**Figure 3:**
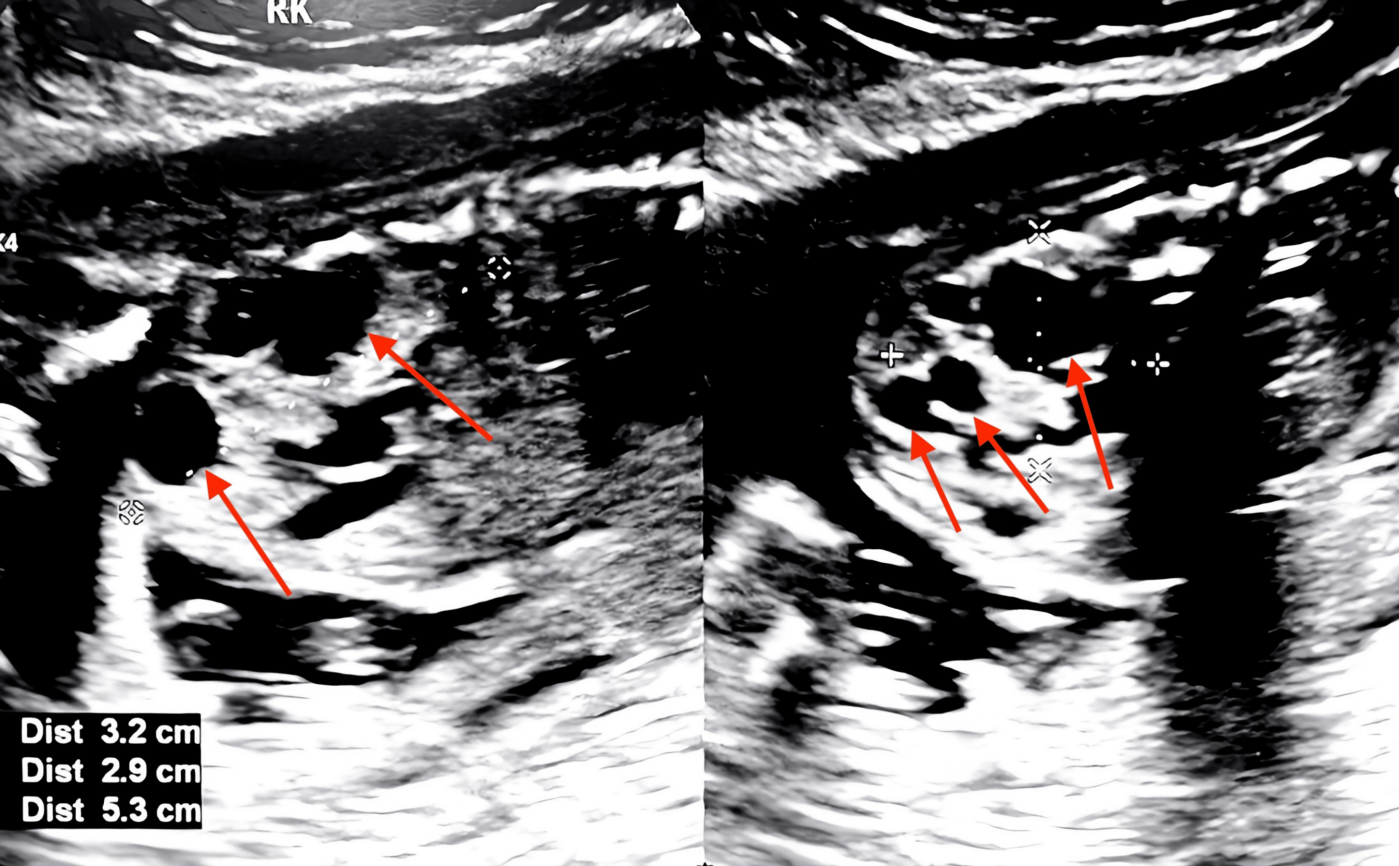
Fetal ultrasound scan at 30 weeks and five days of gestation showing the right multicystic kidney

At 30 weeks and five days, another fetal ultrasound was performed. The fetus was found to have Intrauterine growth restriction (IUGR) with EFW of 1407g (3 lbs, 2oz) at the ninth percentile and AC at the 25th percentile. Normal UA and MCA Doppler were noted. The enlarged right multicystic kidney was still seen (Figure 3), with the largest cyst measuring 6.7 x 5.0 x 5.0 cm. Multiple dilated bowel loops and a normal bladder and sphincter were also observed. The ultrasound also showed female genitalia with enlarged labia. Fetal echo was also normal at this time, with no sign of coarctation of the aorta. The patient was scheduled with a pediatric genetic physician for long-term follow-up.

She was seen at the MFM clinic again at 33 weeks and 35 weeks with fetal ultrasound evaluations. The results were consistent with previous ultrasound readings. Delivery was at 39 weeks. Neonatal intensive care unit (NICU) consult, neonatal renal ultrasound, and echocardiography were performed at birth before discharge. The neonate presented with female genitalia and normal echo readings. Renal ultrasound still showed enlarged kidneys, for which the infant was referred for follow-up with the pediatric urologist and further follow-up with the pediatric genetic physician. However, we do not have the details of these visits. 

## Discussion

Congenital malformation of the renal/urinary system is found in approximately 30-40% of Turner syndrome cases. This renal/urinary malformation increases the risk of urinary tract infection in Turner syndrome neonates. Common renal/urinary tract abnormalities found in Turner’s syndrome include the horseshoe kidney system, duplex collecting system, complete absence of one kidney, crossed ectopia, and pelvic kidney [3]. In the fetus in our case, we observed a multicystic dysplastic kidney, which led to further NIPT testing and amniocentesis that revealed Turner syndrome diagnosis. Multicystic dysplastic kidney is found in 0.2-1/1000 live births, and it is the most common renal abnormality diagnosed prenatally. It is often associated with other abnormalities in about 5-48% of the cases [4].

Turner syndrome is often common in pregnancies that do not make it to term due to miscarriages/stillbirths. For those that do make it to birth, it has a prevalence of 1:2000 to 1:3000. Turner syndrome’s most common chromosomal presentation is 45 XO in half of the cases. It could equally present with partial deletion of the X chromosome or rearrangement of the X chromosome. Another possible presentation is mosaicism Turner syndrome, which only affects some cells. Most cases of Turner syndrome mosaicism are not inherited but rather because of postzygotic nondisjunction of sex chromosomal cells. In the case of mosaic Turner syndrome, the abnormal cell division takes place in early fetal development. A normal 45 XO/46 XY mosaicism does not always result in Turner syndrome. It has phenotypic variation from the typical Turner syndrome to an ovotesticular disorder of sex development with genital ambiguity to normal male phenotype with infertility [5]. The typical 45 XO/46 XY phenotype occurs in 10-12% of Turner cases [6]. In this case, the fetus was found to have a Turner mosaicism of 45 XO/46 XY with 56% female genotype and 44% 46 XY male genotype. Fetal ultrasonography showed that the fetus had female genitalia with enlarged labia.

A significant difference exists between 45 XO/46 XY mosaicism cases diagnosed prenatally versus postnatally. It has been shown that 90% of prenatally diagnosed cases of 45 XO/46 XY show a male phenotype. The current case thus falls among the 10% of the 45 XO/46 XY mosaicism, resulting in a Turner phenotype [7]. Postnatally diagnosed cases are noted to mostly present with genital ambiguity [8]. Regarding phenotypic expression, there are numerous males with 45 XO/46 XY who are not Turner cases but exhibit Turner phenotype. The current case did not express much Turner phenotype.

Further phenotypic evaluation will be needed to examine for short stature and other anomalies as the child grows. In another case report, similar to the present case, the infant had Turner syndrome mosaicism but had genital ambiguity [9]. The child was also known to develop Duchenne muscular dystrophy at about three years of age.

Once a case of Turner syndrome is diagnosed prenatally, it is essential to do a lot of follow-up. Patients diagnosed with Turner syndrome should undergo an echocardiogram with views of the aortic arch, cytogenetic studies to check for Y chromosome material that could increase the risk of gonadoblastoma, a kidney ultrasound, and be referred to an endocrinologist for future sex hormone replacement and growth hormone therapy. Furthermore, Turner syndrome mosaicism with a Y chromosome has been shown to have an increased risk of germ cell tumors like gonadoblastoma and dysgerminoma. In a cohort study that examined cancer incidence in women with Turner syndrome, it was discovered that the Y-chromosome lineage developed gonadoblastoma of the ovary by 25 years in the group with a cumulative risk of 7.9% [10]. To deal with the possibility of cancer, prophylactic gonadectomy is recommended for patients with Turner syndrome mosaicism containing Y chromosome material. 

## Conclusions

This case report illustrates the complexities involved in the prenatal diagnosis and management of Turner syndrome mosaicism, specifically the 45 XO/46 XY karyotype. Initially suspected of having trisomy 22 based on NIPT, the diagnosis was refined to Turner syndrome mosaicism through amniocentesis and FISH analysis. The patient's fetus, presenting with female genitalia and a multicystic right kidney, highlights the phenotypic variability and the need for tailored prenatal and postnatal care. This case underscores the importance of multidisciplinary follow-up involving pediatric endocrinologists, urologists, and genetic counselors to manage the diverse clinical manifestations and associated risks, such as gonadoblastoma. The proactive approach of recommending prophylactic gonadectomy due to the presence of Y chromosome material is crucial for mitigating the risk of malignancy. Future research should focus on the long-term outcomes of individuals with mosaic Turner syndrome, particularly those with the 45 XO/46 XY genotype, to further refine management strategies and improve patient care.
